# Effects of Individualized Centrifugation Training on Orthostatic Tolerance in Men and Women

**DOI:** 10.1371/journal.pone.0125780

**Published:** 2015-05-28

**Authors:** Nandu Goswami, Joyce Evans, Stefan Schneider, Melanie von der Wiesche, Edwin Mulder, Andreas Rössler, Helmut Hinghofer-Szalkay, Andrew P. Blaber

**Affiliations:** 1 Medical University of Graz, Institute for Physiology, Graz, Austria; 2 University of Kentucky, Lexington, United States of America; 3 German Sports University, Cologne, Germany; 4 Faculty of Science, Health Education and Engineering, University of Sunshine Coast, Marrochydore, Queensland, Australia; 5 Institute of Aerospace Medicine, German Aerospace Center (DLR), Cologne, Germany; 6 Simon Fraser University, Burnaby, Canada; University of Bari Aldo Moro, ITALY

## Abstract

**Aims:**

Exposure to artificial gravity (AG) at different G loads and durations on human centrifuges has been shown to improve orthostatic tolerance in men. However, the effects on women and of an *individual-specific* AG training protocol on tolerance are not known.

**Methods:**

We examined the effects of 90 minutes of AG vs. 90 minutes of supine rest on the orthostatic tolerance limit (OTL), using head up tilt and lower body negative pressure until presyncope of 7 men and 5 women. Subjects were placed in the centrifuge nacelle while instrumented and after one-hour they underwent either: 1) AG exposure (90 minutes) in supine position [protocol 1, artificial gravity exposure], or 2) lay supine on the centrifuge for 90 minutes in supine position without AG exposure [protocol 2, control]. The AG training protocol was individualized, by first determining each subject’s maximum tolerable G load, and then exposing them to 45 minutes of ramp training at sub-presyncopal levels.

**Results:**

Both sexes had improved OTL (14 minutes vs 11 minutes, p < 0.0019) following AG exposure. When cardiovascular (CV) variables at presyncope in the control test were compared with the CV variables at the same tilt-test time (*isotime*) during post-centrifuge, higher blood pressure, stroke volume and cardiac output and similar heart rates and peripheral resistance were found post-centrifuge.

**Conclusions:**

These data suggest a better-maintained central circulating blood volume post-centrifugation across gender and provide an integrated insight into mechanisms of blood pressure regulation and the possible implementation of in-flight AG countermeasure profiles during spaceflights.

## Introduction

Post-spaceflight orthostatic intolerance is a common phenomenon in both male and female astronauts. Artificial gravity (AG) administered during spaceflight may prevent the development of orthostatic intolerance upon return to Earth. Ground-based studies observed that AG: a) improved orthostatic tolerance in ambulatory men [1–2]; and b) maintained orthostatic tolerance in bed rested men [3]. In these studies different gravity (G) exposures were administered and for different durations.

The beneficial effects of AG exposure for women are, however, not clear. To our knowledge only two studies have previously examined the effects of AG exposure across gender [4–5]. Fong et al found that only 1 in 6 females could complete their AG protocol [4]. Stenger et al. concluded that three weeks of daily AG training was more effective in men than women with a greater response from subjects who exercised during AG sessions than in those who passively rode the centrifuge [5].

A limitation of the previous studies was, however that all subjects underwent identical protocols. However, it is obvious from these cited studies [4–5] that some subjects are more tolerant to AG protocols than others. In less tolerant subjects, for instance, an identical G load may lead to presyncope and hence premature termination of the AG protocol. As such, it seems reasonable to suggest that AG training profiles should be individualized; tailored to the tolerance capacities of the individual exposed to AG. This would ensure that each subject obtained a tolerable AG training; and that the relative G load and the duration of training would be comparable across all individuals.

In this study we examined the effects of 90 minutes centrifugation (AG) compared to 90 minutes supine on the centrifuge without AG exposure on a subsequent test of the orthostatic tolerance limit (OTL) of men and women. The OTL was determined from the combination of head up tilt (HUT) and lower body negative pressure (LBNP) required to induce presyncope [6–8]. We hypothesized that an individualized AG training program (see methodology below) with an acute ~90-min bout of AG exposure would increase the OTL.

## Methodology

### Subjects

7 males and 5 females (age: 20–45 yrs, height: 160–188 cm, BMI: 20–26, Table 1) were recruited by the Biomedical Science Support Centre within the Institute for Aerospace Medicine at the German Space Centre (DLR). All subjects were physically active, healthy, non-smoking and normotensive individuals. Before being enrolled into the study, all subjects underwent a medical screening which consisted of: clinical-chemical analysis (glucose, creatinine, urea, uric acid, liver enzymes, total cholesterol, high density lipoprotein (HDL) and low density lipoprotein (LDL)); hematology (blood count); urine analysis (glucose, protein, urobilinogen); resting electrocardiogram (ECG); exercise test to verify endurance capacity; a standing test for orthostatic tolerance assessment; and, a medical history. Female volunteers additionally had to test negative on a standard pregnancy test before each experiment.

**Table 1:** Subject characteristics at study start (mean ± sd).

	Male (n = 7)	Female (n = 5)
Age (yr)	27.7±4.2	27.6±4.4
Height (cm)	180.0±4.5	169.8±5.0
Weight (kg)	78.6±5.9	69.6±6.6

All subjects gave their written informed consent after being informed about the potential risks involved. The written consent forms are stored at the DLR. The study was conducted in accordance with ethical principles stated in the Declaration of Helsinki, and the study was approved by the ethics commission of the Northern Rhine medical association (Aerztekammer Nordrhein), Düsseldorf, Germany. Prior to testing each subject was familiarized with all aspects of the study, including the equipment and the personnel involved.

### Experimental Protocol

All experiments were carried out from 8 am to 2:30 pm and were performed at least 2 hrs after a light meal and at least 12 hrs after the last caffeinated or alcoholic beverage. The experiments were conducted at the DLR in Cologne. Room temperature and humidity levels were controlled at 23°C, and 55%, respectively throughout the experiment and during testing the room lights were dimmed.

In this randomized, cross-over study, subjects were placed in the centrifuge nacelle while instrumented and after lying supine on the centrifuge for one hour, they underwent either: 1) AG exposure (90 min) in the supine position [protocol 1, artificial gravity exposure], or 2) lay on the centrifuge for 90 min in the supine position without AG exposure [protocol 2, control]. The spacing of the test protocols was 28 days to avoid effects of menstrual phase on cardiovascular regulation [9] and orthostatic tolerance in women. Equal numbers of subjects received protocols 1 and 2 first.

Following the AG or control phase, subjects were transported on a gurney to an adjacent room where the HUT + graded LBNP was carried out and the orthostatic tolerance time determined. Orthostatic tolerance time was calculated as the time from the onset of HUT to the development of presyncopal symptoms.

### Intervention (Artificial Gravity Training) Day Protocol

On the intervention day subjects were positioned supine on one of the two bed nacelles of the short arm human centrifuge (SAHC) where instrumentation of the subject took place. Participants were instructed to breathe normally and avoid leg movements. After one hour the AG exposure started with baseline level 0 Gz for ten minutes (centrifuge not running), followed by ten minutes of 0.6 Gz acceleration (measured at heart level) for women and 0.8 Gz for men. The acceleration was then increased by 0.1 Gz every three minutes. This continued until the participant experienced pre-syncopal symptoms or the cardiac output dropped to half of the outset value. The subject was then returned to the baseline (0 Gz) level for ten minutes.

**Fig 1:**
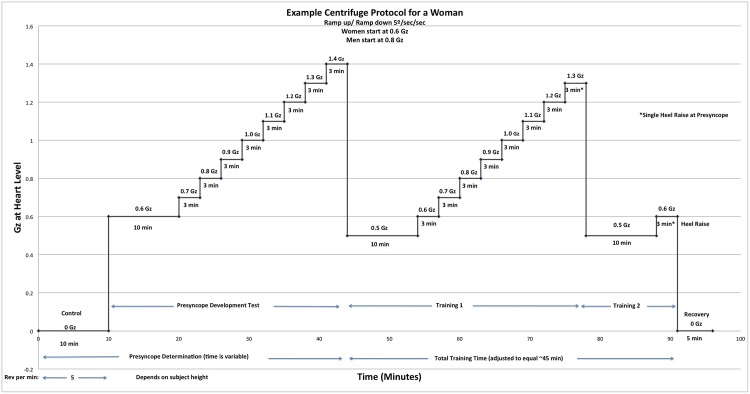
Example of one subject’s individualized AG protocol, consisting of determination of the G level required to produce presyncope (~45 min) followed by a training period, that reached a maximum G level one step below that required to produce presyncope. Training cycles were repeated for a total training period of 45 min. Males and females had different starting g loading (at the level of the heart).

This presyncopal development test was then followed by the AG training run, consisting of several AG ramp increments for a total of 45 minutes (Fig 1). That is, if the subject showed presyncopal symptoms at 1.4 Gz, at the heart, the training level went one level below (that is, 1.3 Gz in this example). The AG training protocol was individualized for each subject.

The first training run started 0.1 G lower than the first value of the presyncopal development test, (that is, at 0.5 G for women and at 0.7 G for men) and lasted for 10 minutes. The acceleration was then increased by 0.1 G, every 3 minutes up to a level of 0.1 G below the maximum tolerance level. At the end of the highest level of the training ramp, independent of presyncopal symptoms, each subject performed a heel raise to support venous return. The acceleration was then reduced to the initial value of the first training run for 10 minutes, and the sequence was repeated until 45 minutes of AG exposure were reached. This protocol ensured that subjects did not reach presyncope during their individualized training. In summary, though the G load and hence the time needed to reach presyncope varied in each subject, training duration was consistent across subjects.

The subject was then transported to the tilt table in the supine position. The transition time from centrifuge to the tilt table, including supine baseline measurements, was standardized to 30 minutes. After instrumentation hook up and supine data collection on the tilt table, the table was tilted to 70° HUT for 5 min. Following this, lower body negative pressure LBNP was applied, starting at -20 mmHg for 3 minutes. The LBNP was then raised by -10 mmHg increments every 3 minutes until the subjects showed presyncopal symptoms. Orthostatic tolerance time was thereby calculated as the time taken from the onset of HUT to the development of presyncopal symptoms. Unless the subject directly requested to stop the test, termination of the HUT-as well as the preceding AG exposure- were determined by an independent medical monitor, who continuously assessed subjects for signs of presyncope (see [6–8]). These signs could be subjective (grey out, light-headedness, dizziness, omnidirectional vertigo, sudden sensation of heat, nausea, sweating) or objective (a systolic blood pressure drop-off > 15 mmHg, and/or a sudden decline in heart rate > 15 bpm, or a sustained tachycardia > 1 min > 90% maximum age-predicted heart rate).

### Control Day (No Artificial Gravity Exposure) Protocol

On the control day, subjects were positioned supine on one of the two bed nacelles of the SAHC where instrumentation of the subject took place. Participants were instructed to breathe normally and avoid leg movements. After one hour, they were not exposed to AG, but continued lying horizontal for the duration of the AG training protocol (which included safety checks, urination etc.); this period lasted approximately 90 minutes. In all other aspects both protocols were identical.

### Physiological Measurements/Monitoring

Subjects were instrumented to obtain continuous beat-by-beat heart rate (HR) by a 3-lead electrocardiogram (Biopac Systems, Goleta, CA, USA) throughout the entire experiment. Continuous beat-by-beat arterial finger blood pressure (BP) was measured with a Finometer device (Finapres Medical Systems (FMS), Amsterdam, The Netherlands). The finger cuff was placed around the third finger of the left hand and the left hand was fixed by a sling at the level of the fourth intercostal space at the assumed level of the heart. Finometer measurements were validated with absolute arterial BP measurements obtained by an automated sphygmomanometer (Intellivue MMS X2, Philips, Best, The Netherlands). Stroke volume (SV) was estimated from the arterial BP waveform using the Modelflow method and Beatscope software (TNO-TPD, Biomedical Instrumentation, Amsterdam, The Netherlands), which incorporates age, weight, height, and gender as previously described in detail [10].

Finally, as AG-induced changes in blood pressure depend on body fluid changes and redistribution, and are influenced by adrenergic neural mechanisms, sympathetic activity was estimated using power spectral analysis of beat-by-beat blood pressure variations (BPV) by measuring low frequency power around in 0.1 Hz. Changes in this power, a surrogate to understand adrenergic neural mechanisms in AG-induced improvement of the OTL with regard to gender difference, was analyzed.

### Statistical Analysis

A mixed model cross over design with gender nested within conditions was used to compare cardiovascular variables during the orthostatic tolerance test in both AG conditions. The change in presyncope time between control and centrifuge protocols was tested against a statistical mean of zero using a Wilcoxon ranked non-parametric test for both male and female subjects. The Wilcoxon rank test was also used to determine if the change in presyncope time was different between male and female subjects.

## Results

### Orthostatic Tolerance Time

**Fig 2:**
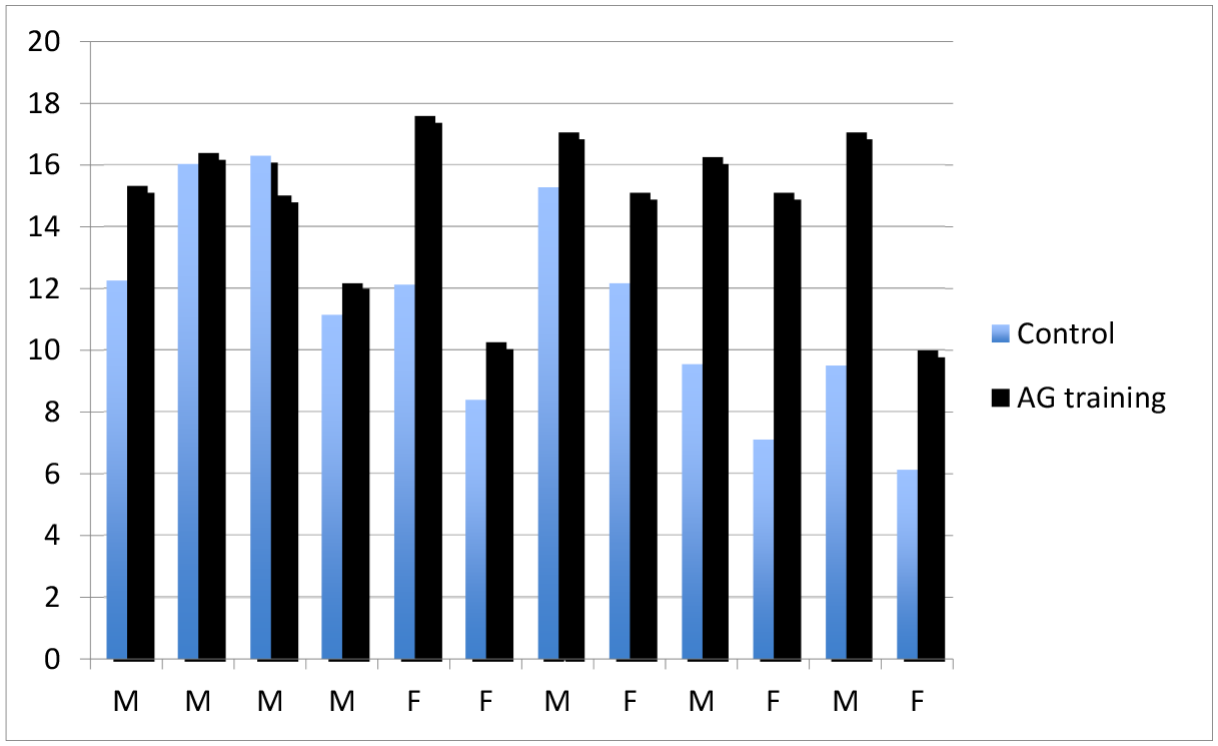
Orthostatic tolerance times (in minutes) as determined on the control day and the AG exposure day.

Each subject was able to complete the 90 minutes training protocol. We observed that AG improved orthostatic tolerance to 14.9 ± 0.7 minutes from 11.6 ± 1.0 minutes (p < 0.0019) and that this improvement was seen in both males and females (Fig 2). Female subjects had a 53% increase in their tolerance (4.47 ± 1.12 min, p = 0.031) and males a 23% increase (2.51 ± 1.12 minutes, p = 0.039). There was no statistically significant difference in the increase in tolerance in females compared to males.

### Cardiovascular Effects

There were no cardiovascular differences at baseline (supine) on control and AG days (Table 2); however, females had lower systolic blood pressure (SBP) (p = 0.033), SV (p = 0.002) and cardiac output (CO) (p = 0.021) compared to males. Compared to baseline, blood pressure (p<0.001), SV (p<0.001), CO (p<0.001) and left ventricular ejection time (LVET) (p<0.001) were all reduced at presyncope while HR (p<0.001) and total peripheral resistance (TPR) (p = 0.021) were elevated (Table 2). At the time of presyncope, HR, SBP, SV, TPR and LVET were similar on control and centrifuge days; however, post-AG presyncope occurred at higher diastolic blood pressure (DBP) (p = 0.047), mean arterial pressure (MAP) (p = 0.032) and CO (p = 0.009) than post control presyncope (Table 2). The increase in TPR from baseline to presyncope was also greater in female (p = 0.040) compared to male subjects in both control and AG conditions.

**Table 2:** Cardiovascular changes from supine baseline (Base) to presyncope (Presync) during 70° head-up tilt with lower body negative pressure following bedrest (control) or centrifuge training (Centrifuge).

Cardiovascular variables	control				Centrifuge						
	male		female		male			female			
	Baseline	Presyncope	Baseline	Presyncope	Baseline	*Isotime*	Presyncope	Baseline	*Isotime*	Presyncope	Main Effects
**HR (bpm)**	71 ± 7	109 ± 7	70 ± 8	130 ± 8	70 ± 7	***109 ± 11***	125 ± 7	75 ± 8	***116 ± 12***	145 ± 8	*
**SBP (mmHg)**	139 ± 5	75 ± 5	108 ± 6	64 ± 6	128 ± 5	***120 ± 5***	82 ± 5	116 ± 6	***120 ± 6***	82 ± 6	* † #
**DBP (mmHg)**	67 ± 4	47 ± 4	55 ± 4	45 ± 4	65 ± 4	***73 ± 4***	52 ± 4	62 ± 4	***75 ± 4***	57 ± 4	* ‡ #
**MAP (mmHg)**	86 ± 4	55 ± 4	72 ± 5	50 ± 5	83 ± 4	***86 ± 4***	61 ± 4	81 ± 5	***88 ± 5***	64 ± 5	* ‡ #
**SV (mL)**	108 ± 5	42 ± 5	85 ± 6	21 ± 6	108 ± 5	***58 ± 4***	44 ± 5	86 ± 6	***40 ± 5***	24 ± 6	* † #
**CO (mL/min)**	7.48±0.47	4.54±0.47	5.96±0.56	2.52±0.56	7.44±0.47	***6*.*71±0*.*42***	5.35±0.47	6.46±0.56	***4*.*54±0*.*45***	3.54±0.56	* † ‡ #
**TPR (mmHg·min/L)**	0.70±0.10	0.74±0.10	0.76±0.12	1.13±0.12	0.70±0.10	***0*.*78±0*.*12***	0.71±0.10	0.76±0.12	***1*.*21±0*.*13***	1.23±0.12	* † ‡

In all but one subject presyncope occurred earlier in the control condition and cardiovascular data at the equivalent time of presyncope in the control test during centrifugation are presented in the “isotime” columns. Baseline: last 2 minutes average prior to head-up tilt. Presyncope: last 5 beats average prior to the termination of the tilt protocol. Isotime: cardiovascular data from the in the centrifuge protocol: average of 5 beats from the same time as presyncope in the control protocol. Significant (p<0.05) main effects:

*, Presyncope vs. baseline;

†, Female vs. male;

‡, centrifuge vs. control;

#, Isotime centrifuge vs. control presyncope.

**Fig 3:**
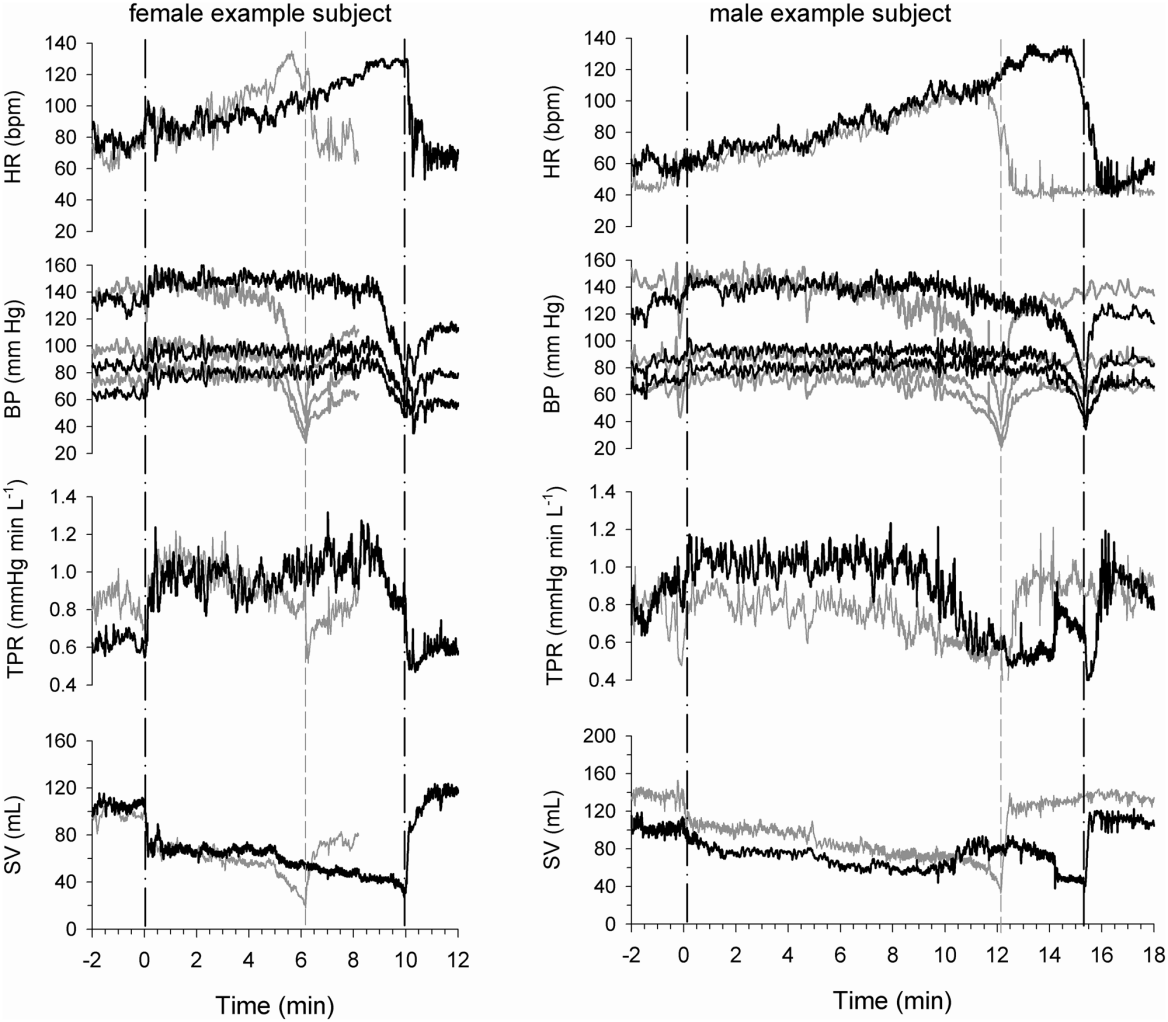
Cardiovascular responses to head-up tilt with graded LBNP to presyncope. An example of a female subject is shown on the left (Fig 3a) with a male subject on the right (Fig 3b). Physiological data (HR: heart rate; BP: blood pressure; TPR: Total peripheral resesistance; SV: Stroke volume) are shown from 2 minutes prior to tilt until 2 minutes recovery from tilt; grey represent data from the control day and data shown in black represent data on the artificial gravity (AG) day. Tilt started at time zero, and the times of presyncope, are shown by vertical dashed lines in grey (*control*) and black (*AG protocol*), which also represents *isotime* (as utilized in Table 2).

To further elucidate factors associated with the increased time to presyncope on the AG day, presyncopal data from the control protocol were compared with post-centrifuge orthostatic tolerance test data, that corresponded with the control time of presyncope (*isotime*). An example of this comparison is shown in Fig 3, which includes a typical tracing of heart rate, blood pressure, total peripheral resistance and stroke volume in a female (Fig 3a) and a male (Fig 3b) as they reach presyncope on both control and AG days.

Compared to control day presyncope, istotime blood pressure (p<0.001), SV (p = 0.004) and CO (p = 0.001) were all greater on the AG day. Although TPR was not different between presyncope and isotime, the difference between the TPR of men compared to women was more pronounced at isotime than observed at presyncope (p = 0.008).

Both HRV-LF (p = 0.0002) and HRV-HF (p = 0.0009) were reduced 5 minutes prior to presyncope compared to baseline. This response to presyncope was independent of gender and type of pre-conditioning. Similarly, both BPV-LF (p<0.001) and BPV-HF (p<0.001) were increased 5 minutes prior to presyncope independent of gender and prior pre-conditioning.

## Discussion

The innovative aspect of the present study is the incorporation of an individualized training protocol, based on each person’s presyncopal tolerance level. We observed that individualizing the AG training protocol not only allowed women to complete the training session, but it also improved orthostatic tolerance times in both men and women in a subsequent stand test. We are not aware of any study that previously examined the effects of individualized AG training on orthostatic tolerance across gender.

Previous studies, which reported the effects of AG exposure, have used identical profiles for subjects who had different tolerance levels. In 2004 and 2007 studies, the usefulness of AG training to improve OTL was demonstrated [1,5]. These studies tested the OTT of men [1,5] and women [5] before and after a three weeks’ AG training. Over these 3 weeks, every participant was exposed to artificial gravity for 35 minutes a day. After an initial 7- minute warm-up run at 1G (at the foot), acceleration was increased to 2.5G for 2 minutes and then returned to 1G for 2 minutes. This cycle was repeated for a total of 35 minutes of daily training. The participants were randomly divided into active (performing bicycle ergometry during AG exposure) or passive (no exercise) groups. The orthostatic tolerance time was determined by a head-up tilt with lower body negative pressure applied, once before and once after the three training weeks. The results showed that men, especially those doing exercise, greatly benefitted from the AG training. The female participants with exercise also benefitted, though less than men, while females without exercise showed no improvement at all. When combining the active and passive groups, women showed no significant improvement. The authors suggested that a possible reason for this might have been due to location of the interface between the LBNP chamber seal and the body: the female participants tended to have a larger part of their splanchnic region subjected to chamber pressure. In a previous study in which we examined the effects of the placement of the LBNP seal at different positions, we showed that inclusion of the splanchnic region in the LBNP chamber led to different cardiovascular responses [11]. Furthermore, the splanchnic region can play an important role in cardiovascular regulation during central hypovolemia induced by LBNP [12–13]. Therefore, based on results from the previous study of Stenger et al [5] it is difficult to make a conclusion on the beneficial effects of AG.

Another recent study showed similar results: Fong et al tested 6 men and 5 women in a single 60 minutes trial on a short-armed human centrifuge [4]. Again, every participant underwent the same protocol: all starting from a sustained 2.5G (at the feet), regardless of their individual constitution or whether they were male or female. The protocol proved to be a severe challenge to many of the subjects with several failing to complete the 60-minute training duration. There was a gender bias: one of six men did not complete the trial, while in the women’s group, four of the five females could not withstand the AG exposure for the full 60 minutes. For these subjects the experiment had to be stopped prematurely due to the development of presyncope.

These previous studies indicate that AG training improved OTT in men with little to no effect in women [4–5]. The results also suggest that women had reduced tolerance to AG compared to men; a common observation (reviewed recently in [14–15]). The fact that women have lower tolerance dictates earlier sensation of dizziness/ fainting in training, which implies that with a fixed G protocol the training duration should differ between men and women and would prevent direct gender comparisons. To eliminate this G-exposure time inequality we designed a protocol based on that of Iwase [2]. Similar to Iwase, G-training in our study was individualized to match each subject’s presyncopal endpoint. AG-training started with a presyncopal development test, aimed at determining each participant’s individual G tolerance level. Subsequently, we exposed each subject to a G-load referenced to this G tolerance level, and subsequently, each training session lasted 45 minutes and was conducted at a G load just below that person’s presyncopal G load. Our results in men confirm the previously reported results of Iwase et al [2]. To our knowledge, we are the first to extend this principle of individualized AG training to women. Hence, this protocol design allows the results to be more accurate as it removes personal fitness factors. In fact, by doing so, we are the first to report that individualized AG training improves OTT in both men and women.

### Cardiovascular Responses

Our data clearly indicate significant differences between the cardiovascular status of subjects on a day following AG exposure compared to a day following head down bedrest (Table 2, Fig 3). First, differences were not seen at supine baseline on the two days. However, DBP, MAP and CO were higher at presyncope following AG while HR, SBP, SV and TPR were the same; and second, during *isotime*, blood pressure, SV and CO were higher than at presyncope on the control day, with HR and TPR similar on the two days. These data indicate that central blood volume-related hemodynamic parameters were elevated (blood pressure, SV, CO) during orthostatic challenge on the AG compared to the control day, while hemodynamic parameters associated with cardiac (HR) and vascular (TPR) function were similar.

It has been well established that sympathetic control of peripheral vasculature is lower in premenopausal women than men. Reduced vascular control has also been suggested as a contributing factor to syncope and increased incidence of presyncope in returning astronauts. Although we did not measure sympathetic activity directly (i.e. via MSNA), we did collect beat-to-beat blood pressure and heart rate. Both of these indices indicate that there was an increase in sympathetic activation in the 5 minutes prior to presyncope when compared to baseline. There was no indication from these non-invasive measurements that gender or prior centrifugation had any effect on these responses.

We speculate that the possible benefits of AG on orthostatic tolerance could be due to either/ all of the following: changes in splanchnic volume, changes in cardiac contractility, AG induced changes in muscle tone in the lower limbs or capillary filtration in the lower body. Briefly: i) It is possible that the repeated AG challenge near the individual orthostatic tolerance threshold produced an increase in splanchic blood volume redistribution, which could have maintained the blood pressure level. The role of splanchnic volume in arterial blood pressure regulation during central hypovolemia has previously been reported [12–13, 16]; ii) At presyncope cardiac contractility is reduced [7]. Could the AG training lead to increases in cardiac contractility and could this have led to increased orthostatic tolerance? This speculation is also supported by our results that show that heart rate and peripheral vascular resistance were not changed at presyncope, with and without AG training.

In the mid 1990’s Convertino and his group published a series of papers which described the effects of a single bout of maximal cycle ergometry exercise on cardiovascular function and orthostatic tolerance following 16 days of head-down tilt bed rest (HDT-BR) [17–19]. Similar to our study, their protocol was conducted as a randomized, cross-over design with control and exercise conditions, The standardized exercise protocol was performed 24 h before the end of HDT-BR and consisted of a 4-min baseline of 200 kilopond-meters (kpm)/min followed by stepped increase in work rate of 100 kpm/min every min to exhaustion. From these data it was concluded that a single bout of maximal exercise following 16 days HDT-BR could restore orthostatic tolerance (supine LBNP) to pre-HDT-BR levels. Following exercise their subjects had greater circulating levels of norepinephrine, vasoconstriction, cardiac output, baroreflex sensitivity and plasma volume. However, in their study, the fluid intake of the subjects post-exercise was not regulated and it was found that there was a significant increase in fluid intake and reduction in urinary output which was mostly due to an increased thirst reflex and a modified renal tubular reabsorption of sodium. Since an increase in plasma volume was one of the contributors to orthostatic tolerance it could have accounted for a significant portion of the observed increase in orthostatic tolerance. In a subsequent experiment without HDT-BR Convertino [20] showed that exercise to exhaustion enhanced α_1_-adrenergic responsiveness within a 24-h period following the exercise. This was found to be in contrast to the effects of repeated bouts of sub-maximal exercise [21]. When taken together these studies suggest that maximal stimulus of the cardiovascular system is required to sufficiently increase vascular, renal and cardiac baroreflex responsiveness to orthostatic challenge.

### Gender differences in orthostatic tolerance

Presyncopal astronauts are different from non-presyncopal ones in that they display lower levels of standing norepinephrine levels and lower total peripheral vascular resistance [22]. Indeed, it is known that presyncopal astronauts also have low peripheral vascular resistance, before and after flight [23].

Women seem to have greater problems with orthostatic instability after spaceflight than men [24]. It is not completely understood what the specific mechanisms are; however, they seem to be multifactorial [22]. In response to lower body negative pressure, to standing, to mental stress and to cold pressor tests, women have smaller increases in vascular resistance than do men [22]. Though any single effect may not necessarily present a problem, combined with other factors such as the graded hypovolemia such as what was applied in our study, can lead to difficulty maintaining standing pressure and consequent orthostatic intolerance.

Waters et al. [23] suggested that the most important factor explaining low vascular resistance in females might be the presence of estrogen. Estrogen has an effect on the endothelium-dependent vasodilation: it augments vasodilation [25]. The vasodilation consequently lowers vascular resistance and venous return.

In the study of Waters et al [23], men who did not suffer from syncope had considerable advantages: They had the highest total peripheral resistance, they maintained their standing stroke volume regardless of plasma volume changes, and they exhibited hyperadrenergic responses on landing day, allowing them to increase resistance and maintain arterial pressure—probably the most important factor.

In our study both men and women showed an increase in OTL following exposure to AG. A principal outcome of this study was the demonstration of the importance of developing an AG protocol tailored to each subject’s tolerance, regardless of his or her level of fitness. Through this, we demonstrate that individualized training is more efficient and has a bigger effect on the individual than a “blanket” AG protocol. This protocol design is relatively new to artificial gravity testing, having been used in men [2] but not women, and allows the results to be more accurate as it removes personal fitness factors.

Our results are of great importance as microgravity induced deconditioning is one of the most significant problems of human spaceflight. Centrifuge training in space, e.g. on the ISS, could have a positive impact on the constitution of returning astronauts. Performing SAHC training in space would potentially increase astronauts’ OTL upon landing, and as such may prevent syncopal symptoms. Further studies will be needed to investigate whether the time span of the AG training could be shortened or otherwise optimized, for instance by augmenting AG exposure with exercise such as bicycle training during the centrifuge run, which is known to increase the tolerance to AG exposure [2], potentially allowing for higher G-loads during training to improve its efficacy.

### Limitations

We could not directly measure cardiac contractility or splanchnic volume changes in this study. Our results, however, do indirectly suggest that the AG could have changed these parameters and they could have affected the orthostatic tolerance. Future AG training studies should examine the role of splanchnic volume changes as well as cardiac contractility and calf blood flow on cardiovascular regulation in subjects reaching presyncope.

To analyze adrenergic neural mechanisms in AG-related improvement of the OLT, the plasma level of noradrenaline is an important indicator to demonstrate changes in adrenergic neural function induced by AG. We did not measure noradrenaline during head-up tilt or centrifugation. We did observe a significant increase in HR and an increase in the ratio LF/HF HF/LF (Table 3), which is considered an indicator of a greater reliance on sympathetic over parasympathetic control of the heart. Furthermore the increase in total BPV including the LF region would suggest an increase vasomotor activity associated with sympathetic control of peripheral resistance vessels.

**Table 3:** Heart rate variability (HRV) and blood pressure variability (BPV) changes during five minutes of supine baseline and 5 minutes prior to presyncope during head-up tilt following either a supine control period or centrifuge pre-conditioning.

	Control				Centrifuge			
	Male		Female		Male		Female	
	baseline	presync	baseline	presync	baseline	presync	baseline	presync
**HRV**								
LF	2260±580	432±580	1070±690	1660±690	2250±580	330±580	2690±690	167±690
HF	2480±830	210±830	2150±980	1850±980	2140±830	120±830	2730±980	90±980
LF/HF	1.43±0.59	2.84±0.59	1.23±0.70	2.24±0.70	1.72±0.59	2.97±0.59	1.76±0.70	3.14±0.70
**BPV**								
LF	9.68±3.00	26.5±3.0	4.21±3.55	17.0±3.6	8.83±3.00	20.4±3.0	6.64±3.55	16.3±3.6
HF	2.80±1.58	11.9±1.6	1.13±1.87	6.06±1.87	2.99±1.58	7.56±1.58	2.33±1.87	8.46±1.87

LF: low frequency (0.04–0.15 Hz) power; HF: high frequency (0.15–0.5 Hz) power; LF/ HF HF/LF: power ratio. HRV power unit is (ms)^2^; BPV power unit is (mm Hg)^2^.

## Conclusions

In the present study, we investigated whether artificial gravity training had a positive effect on the orthostatic tolerance limits of men and women. The hypothesis was tested that short-arm centrifuge training would improve the orthostatic tolerance time of 1hr supine rested men and women. Our results provide an integrated insight into mechanisms of blood pressure regulation, and across gender, using an individualized AG countermeasure. The optimal AG protocol remains to be defined. However, our results are encouraging in that we observed that an individualized training paradigm was more tolerable, and increased OTL in women. Future research should examine whether AG exposure can be optimized to maintain the orthostatic tolerance following spaceflight.

## Supporting Information

S1 File Data for Fig 1. (XLS)

S2 File Data for Fig 2. (XLS)
